# Supplementary figures and images for: Genetic diversity and population structure in the Ryukyu flying fox inferred from remote sampling in the Yaeyama archipelago
Source: PLoS One. 2021 Mar 18;16(3):e0248672. doi: 10.1371/journal.pone.0248672 (PMC7971866; doi:10.1371/journal.pone.0248672)

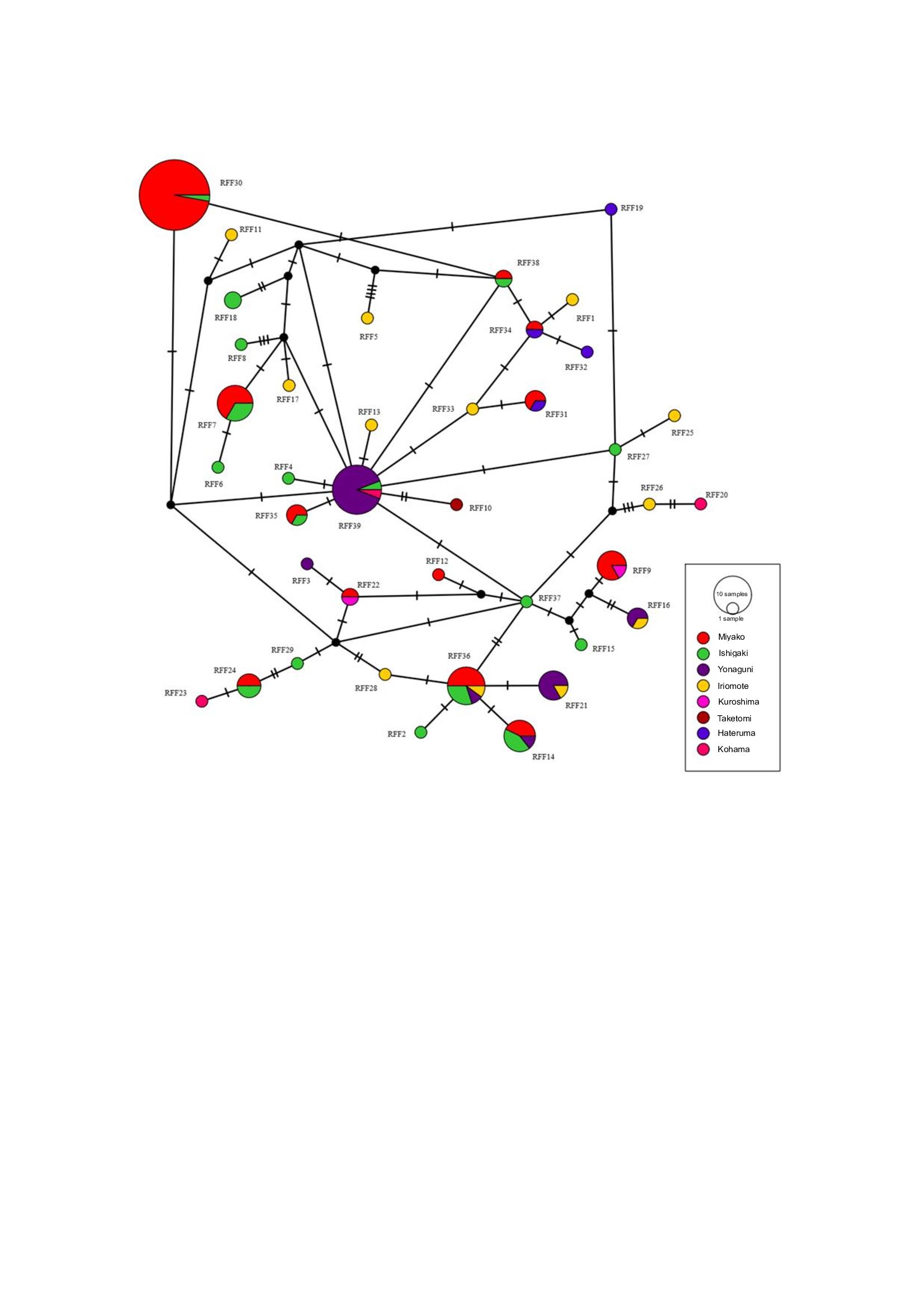

Supplement: S2 Fig — Each haplotype is shown as a circle with ratio of the number of samples of each island. The sizes of circles represent the sample sizes. (TIFF) [file pone.0248672.s002.tiff]

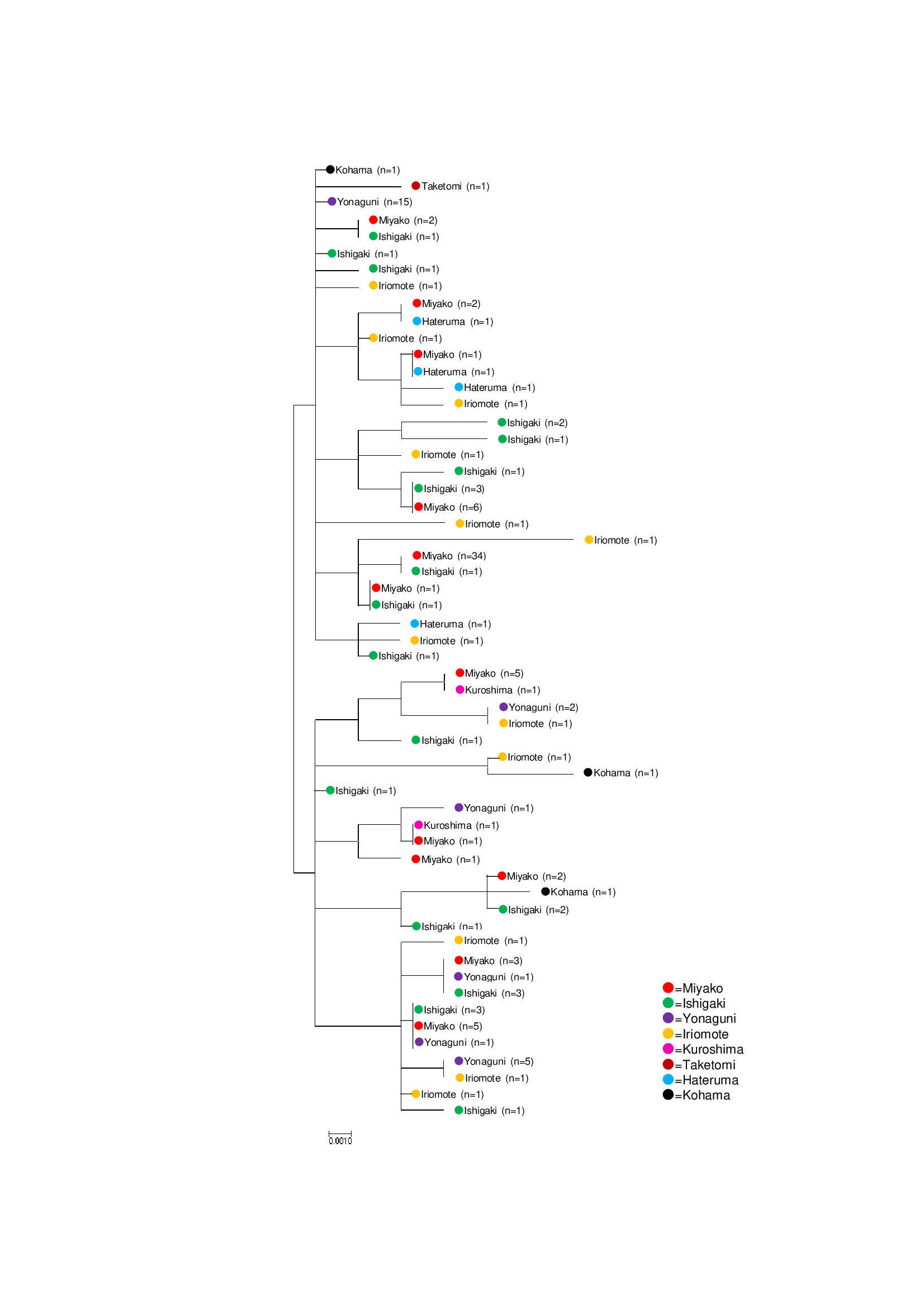

Supplement: S3 Fig — Population and sample number are shown in each place in the tree. (TIFF) [file pone.0248672.s003.tiff]

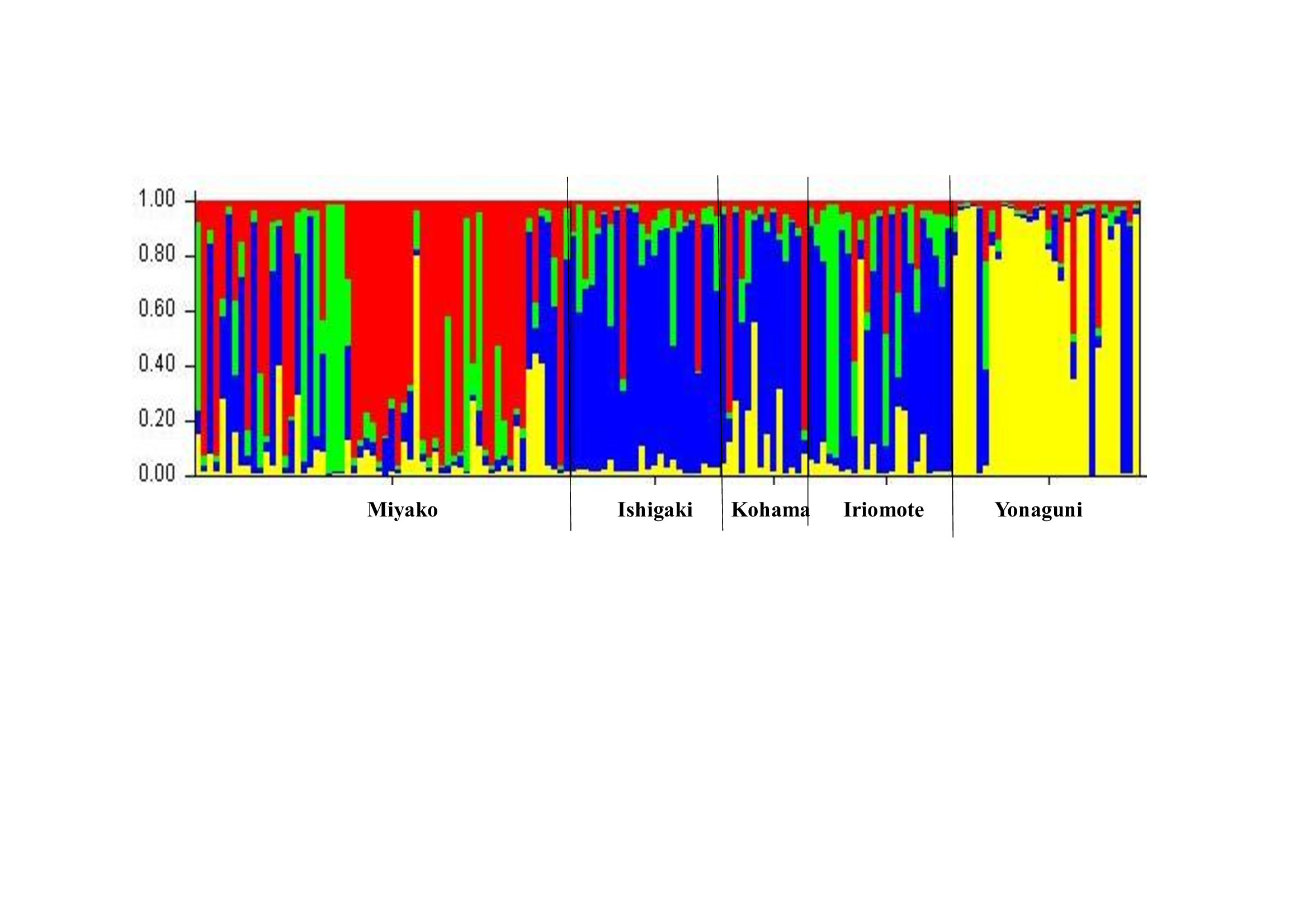

Supplement: S5 Fig — Each ancestral population is shown with colors. (TIFF) [file pone.0248672.s005.tiff]

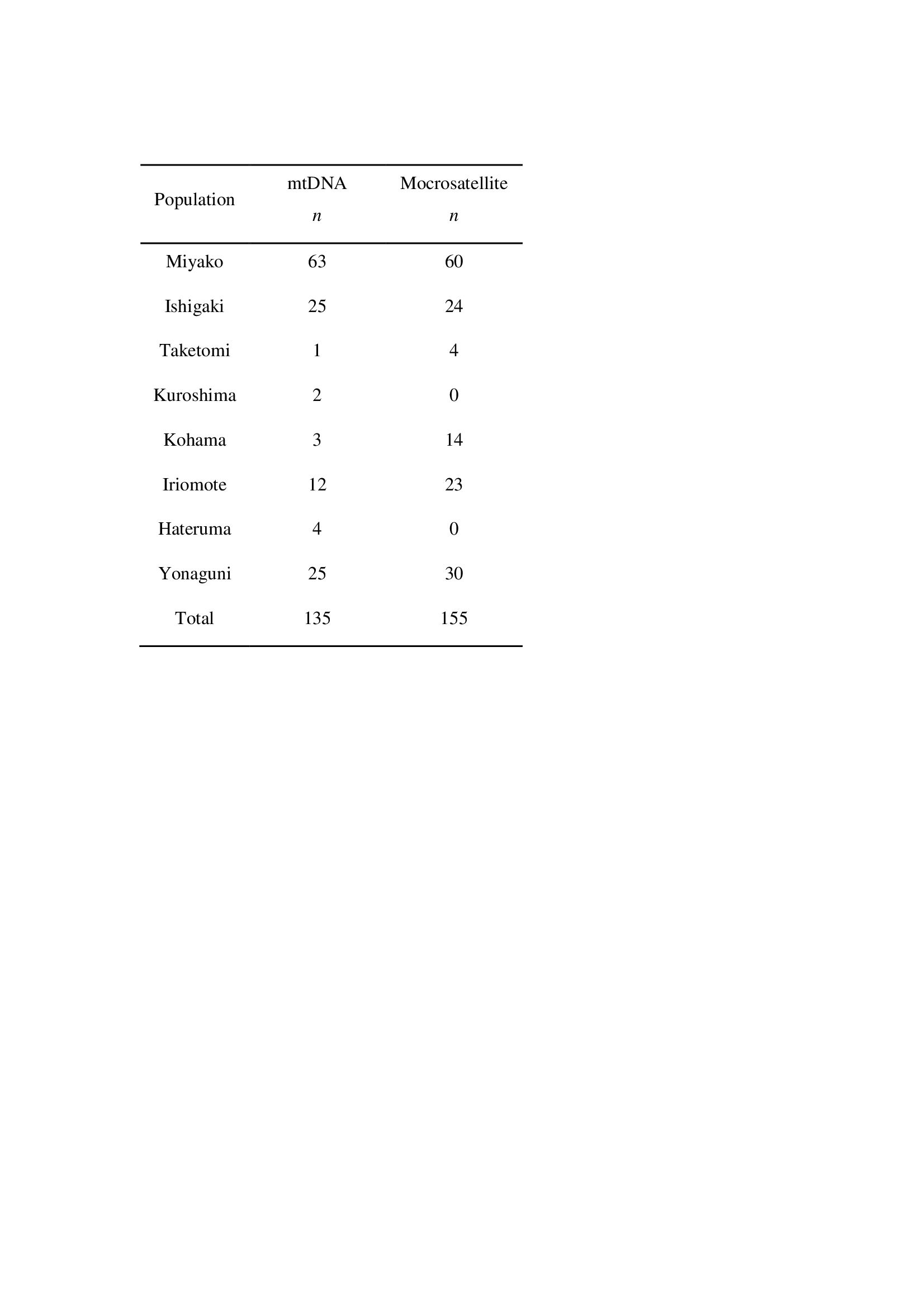

Supplement: S1 Table — (TIFF) [file pone.0248672.s006.tiff]
